# Impact of different doses of rivaroxaban on clinical outcomes in elderly patients with non-valvular atrial fibrillation: a real-world study

**DOI:** 10.3389/fphar.2025.1714318

**Published:** 2025-12-09

**Authors:** Juan Yu, Zufeng Zhang, Qingru Yuan, Haobing Hu, Huimin Li, Meifang Huang, Youlin Mao

**Affiliations:** 1 Department of Cardiology, The Ninth People’s Hospital of Zhengzhou, Zhengzhou, Henan, China; 2 Department of Cardiology, The Seventh People’s Hospital of Zhengzhou, Zhengzhou, Henan, China

**Keywords:** rivaroxaban, non-valvular atrial fibrillation, elderly patients, different doses, real-world study

## Abstract

**Introduction:**

This study to assess the associations between different rivaroxaban doses and clinical outcomes in elderly NVAF patients in a real-world setting, and to explore factors influencing dose selection.

**Methods:**

A retrospective analysis was conducted on elderly patients (aged ≥65 years) diagnosed with NVAF and hospitalized at the Ninth People’s Hospital of Zhengzhou between 1 January 2022, and 31 December 2022. All patients received rivaroxaban therapy. Based on the prescribed dose, patients were categorized into a standard-dose group (15–20 mg/day) and a low-dose group (10 mg/day) groups based on the prescribed dose. Clinical outcomes within 12 months post-discharge were collected via follow-up, including readmission records and telephone interviews.

**Results:**

A total of 214 elderly patients with NVAF were included in the study. The mean age was 79.2 ± 8.2 years in the low-dose group and 75.4 ± 8.0 years in the standard-dose group (P = 0.001). There were no significant differences were observed between the two groups in terms of sex (P = 0.298), CHA_2_DS_2_-VASc score (P = 0.783) and HAS-BLED score (P = 0.586). The low-dose group had a significantly higher proportion of older patients (P = 0.001) and individuals with a history of bleeding (21.42% vs. 7.79%, P = 0.004). Follow-up revealed no statistically significant differences between the two groups in thromboembolic events (P = 0.308) and bleeding events (P = 0.187). However, the standard-dose group had a higher 12-month cumulative survival rate (HR = 0.61; 95% CI: 0.39–0.95; log-rank; P = 0.03). After propensity score matching to balance baseline features, there was no difference between the two groups. Multivariate logistic regression analysis identifiedthe following as significant factors influencing dose selection: age (OR = 0.95, 95% CI: 0.91–0.98, P = 0.004), history of bleeding (OR = 0.36, 95% CI:0.14–0.95, P = 0.038), and estimated glomerular filtration rate (eGFR) (OR = 0.99, 95% CI: 0.97–1.00, P = 0.019).

**Conclusion:**

This retrospective study found that low-dose rivaroxaban was not significantly different from the standard dose in terms of thromboembolic events, bleeding, and all-cause mortality in elderly patients with NVAF. Although the safety profiles appeared similar between the two groups, the standard dose was associated with a higher cumulative survival rate in the unadjusted analysis. However, this observed survival difference may be influenced by baseline confounders, such as age and bleeding history. These findings suggest an association and support the need for further investigation into flexible anticoagulation strategies in clinical practice. Age, bleeding history, and renal function must be carefully considered when individualizing treatment.

## 1 Introduction

Atrial fibrillation (AF) is one of the most common arrhythmias, and its prevalence increases significantly with age. Elderly patients with AF are more than five times as likely to have a stroke as individuals without AF. Epidemiological studies project that, by 2050, there will be approximately 72 million AF patients aged 60 years and older in Asia, with non-valvular atrial fibrillation (NVAF) comprising the majority of cases (Virani SS et al., 2020; Tse HF et al., 2013) The primary clinical concern for patients with AF is the increased risk of thromboembolic events, particularly ischemic stroke. Consequently, clinical guidelines recommend anticoagulation therapy as the cornerstone of stroke prevention in patients with NVAF (Hindricks et al., 2020; Joglar et al., 2023; Arnett DK et al., 2019).

Direct oral anticoagulants (DOACs) are endorsed as preferred alternatives to warfarin (Steffel J et al., 2021; Wang Y et al., 2024; Patel MR et al., 2011). Rivaroxaban, in particular, has a fixed dosage and does not require regular laboratory monitoring; therefore, it has been widely adopted for treating patients with NVAF. Based on findings from the ROCKET AF Phase III Trial, current guidelines recommend a daily dosage of 20 mg of rivaroxaban for patients with creatinine clearance (CrCl) of at least 50 mL/min and a daily dosage of 15 mg of rivaroxaban for patients with CrCl between 30 and 49 mL/min. These recommendations align with the 2024 Chinese expert consensus (Zhao N et al., 2022). However, real-world data indicate that the use of a reduced 10 mg daily dose is significantly more common in Asia than in Europe or North America (Camm AJ et al., 2020). This discrepancy may be attributed to distinctive pharmacokinetic and pharmacodynamic profiles observed in Asian populations. These profiles include lower average body weight, genetic variations in drug-metabolizing enzymes, and a more conservative approach to bleeding risk among clinicians.

Managing anticoagulation in elderly patients with NVAF presents unique challenges. Age-related physiological decline in renal function, which is defined as a reduction in eGFR of at least 3 mL/min/1.73 m^2^ per year, increases the risk of drug accumulation. Additionally, polypharmacy, including the concurrent use of antiplatelet agents and nonsteroidal anti-inflammatory drugs (NSAIDs), increases the risk of bleeding. Frailty and multiple comorbidities (e.g., hypertension and diabetes) further complicate treatment decisions (Yao X et al., 2017; Lau et al., 2022). Importantly, clinical evidence supporting the efficacy and safety of the prevalent low-dose strategy for selected high-risk elderly patients is limited and inconsistent. Existing guidelines offer little guidance on dosage adjustments specific to this population. Consequently, clinicians often empirically reduce the dosage (e.g., to 10 mg daily). This raises a key clinical question: Does reducing the dose sufficiently maintain anticoagulant efficacy while mitigating bleeding risk in frail elderly patients? Long-term safety data in this subgroup remains scarce.

The ROCKET AF trial demonstrated the effectiveness of the standard dose of rivaroxaban. However, the elderly population enrolled in the trial (median age: 73 years) differs significantly from elderly patients in the real world, particularly those aged 80 years and older who are at high risk for bleeding (HAS-BLED score ≥3). Most prior studies have focused on European and North American populations, leaving a gap in the evidence regarding dose optimization for elderly Asian patients, particularly those in China. Results from existing real-world studies on the outcomes of low-dose rivaroxaban are conflicting. Some studies report comparable safety and efficacy, while others suggest an increased thromboembolic risk, highlighting the uncertainty and need for further investigation.

In the context of widespread yet variably justified off-label dosing and unresolved clinical uncertainty, the present study uses a single-center retrospective analysis to address this gap. The study evaluates the impact of low-dose rivaroxaban on key clinical outcomes, including thromboembolic events, major bleeding, and all-cause mortality, in an elderly Chinese patient cohort with NVAF. Additionally, the study seeks to identify clinical factors that influence dose selection, such as age, estimated glomerular filtration rate (eGFR), and bleeding history. The study’s findings aim to inform more personalized, evidence-based anticoagulation strategies for elderly Asian patients with NVAF, particularly those at the highest risk.

## 2 Materials and methods

### 2.1 Study population

This study examined 240 elderly patients diagnosed with NVAF who were hospitalized at the Ninth People’s Hospital between 1 January 2022 and 31 December 2022. The inclusion criteria were as follows: 1) age of at least 65 years and a diagnosis of NVAF; 2) a complete medical history and current use of oral anticoagulation therapy with rivaroxaban (Bayer Pharmaceuticals, Leverkusen, Germany) at a daily dose of 10 or 15–20 mg; and 3) dosing in accordance with the Chinese Expert Consensus on the Diagnosis and Treatment of Atrial Fibrillation in the Elderly (2024 edition).

**FIGURE 1:**
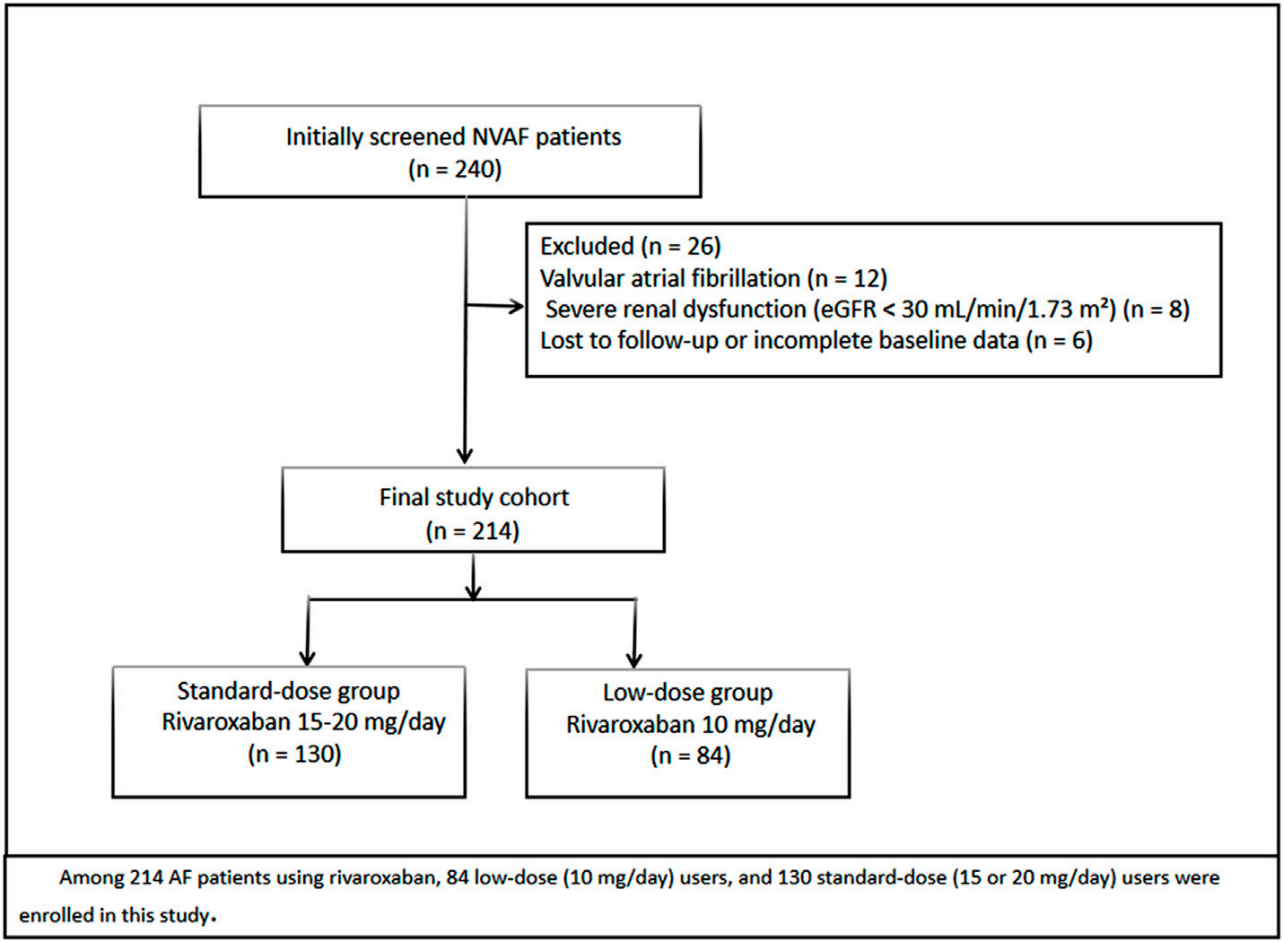
Study flow diagram of patient selection.

The exclusion criteria were as follows: 1) presence of valvular atrial fibrillation, 2) severe hepatic or renal dysfunction (Child-Pugh class B or C or eGFR <30 mL/min/1.73 m^2^), 3) participation in other clinical drug trials, 4) known allergy to rivaroxaban, 5) presence of bleeding disorders or thrombocytopenia. And a total of 240 elderly patients with NVAF were initially screened for eligibility. After applying the inclusion and exclusion criteria, 26 patients were excluded for the following reasons: Twelve had valvular atrial fibrillation, eight had severe renal dysfunction (eGFR <30 mL/min/1.73 m^2^), and six were lost to follow-up or had incomplete data. Thus, the final study cohort comprised 214 patients. Figure 1 depicts the process of patient selection in detail.

Creatinine clearance rates could not be calculated in this study due to a lack of participant body weight data. Therefore, we used the CKD-EPI formula to estimate the glomerular filtration rate (eGFR) based on serum creatinine, age, and gender. These eGFR formulas have been widely recommended by international guidelines, validated in large populations, and are more accurate reflections of true glomerular filtration rates than creatinine clearance rates based on body weight. They also reduce bias due to variability in body size.

This study was conducted in accordance with the Declaration of Helsinki and approved by the Ethics Committee of the Ninth People’s Hospital of Zhengzhou (approval no. LLKY-202422, date: 19 July 2024). Due to the retrospective nature of the study, which entailed analyzing anonymized data from medical records, the Ethics Committee waived the need for obtaining individual written informed consent.

### 2.2 Patient information collection

Patients were categorized into two groups according to the prescribed rivaroxaban dosage: the low-dose group (10 mg/day) and the standard-dose group (15–20 mg/day). Current international and Chinese guidelines recommend both 15 mg and 20 mg doses as standard, full therapeutic options for Patients with NVAF, primarily determining the selection based on renal function (with the 15 mg dose intended for patients with moderate renal impairment and the 20 mg dose for those with preserved renal function). In our cohort, 78 patients received 15 mg daily, and 52 patients received 20 mg daily. A review of medical records confirmed that these prescriptions were consistent with the renal function criteria recommended by the guidelines. To compare the overall strategy of standard full-dose anticoagulation with a reduced 10 mg dose and ensure sufficient statistical power for the analysis, the two dosing regimens were combined into a single comparator group. The CHA_2_DS_2_-VASc score and HAS-BLED score were used to evaluate thromboembolic and bleeding risks.

### 2.3 Clinical outcomes and follow-up

Clinical outcome data were obtained from subsequent hospitalization records or telephone follow-up for all eligible patients. The primary clinical outcomes included.

1. Composite endpoint: All-cause mortality;
2. Primary efficacy endpoint: Thromboembolic events, including stroke, myocardial infarction, and systemic embolism;
3. Primary safety endpoint: Major bleeding events (including intracranial hemorrhage and bleeding requiring transfusion of more than two units of red blood cells);
4. Secondary safety endpoint: Non-major bleeding events (e.g., gastrointestinal bleeding, urinary tract bleeding, gingival bleeding, epistaxis, or bleeding from skin or mucosal surfaces);
5. Secondary endpoint: Cardiovascular-related readmission rate.

All follow-up data were collected by trained healthcare professionals to ensure data accuracy and quality.

#### 2.3.1 Clinical outcome definitions

Primary Efficacy Endpoint (Thromboembolic Events): Thromboembolic events included: Ischemic stroke: A new focal neurological deficit of sudden onset lasting ≥24 h or resulting in death. It must be confirmed by brain imaging (CT or MRI) to be of ischemic origin in accordance with the 2018 AHA/ASA criteria. Systemic embolism: Acute vascular occlusion of an extremity or organ documented by angiography, computed tomography angiography (CTA), or surgical findings. Myocardial infarction (MI): Diagnosed according to the Fourth Universal Definition of MI, requiring a typical rise and fall of cardiac troponin values, with at least one value above the 99th percentile upper reference limit and evidence of myocardial ischemia (e.g., ischemic symptoms, electrocardiogram [ECG] changes, or imaging evidence of new loss of viable myocardium).

Primary Safety Endpoint (Major Bleeding): Defined according to ISTH criteria as fatal bleeding or bleeding at critical anatomical sites (e.g., intracranial or retroperitoneal) or a hemoglobin decrease of at least 2 g/dL. Intracranial hemorrhage was confirmed through neuroimaging.

Secondary Endpoints: Included non-major bleeding events (as defined by ISTH minor bleeding criteria), and cardiovascular-related readmission rate.

#### 2.3.2 Adjudication of clinical events

To ensure objective and accurate endpoint assessment, an independent, blinded Clinical Event Committee (CEC) underwent a centralized adjudication process for all reported clinical events. Two blinded senior cardiologists independently reviewed all source documentation (including medical records, laboratory reports, and imaging studies) for each potential event. Any discrepancies between the initial reviewers were resolved through discussion and, if necessary, arbitration by a third, blinded cardiovascular specialist. Only events that reached final consensus and occurred during the 12-month follow-up period while the patient was on the assigned rivaroxaban regimen were confirmed and included in the analysis.

### 2.4 Data completeness and follow-up

The integrity and completeness of the data were rigorously assessed. Of the 240 patients initially screened, 26 were excluded prior to analysis, primarily due to incomplete baseline data or loss to follow-up. This resulted in a final analytical cohort of 214 patients. This represents an 89.2% (214/240) follow-up completeness rate. Baseline characteristics for the included patients were fully documented, with no missing data for key variables such as age, sex, CHA_2_DS_2_-VASc score, HAS-BLED score, and renal function. Outcome data were systematically collected using a combination of hospital readmission records and structured telephone interviews to minimize the risk of missing events that occurred outside the hospital setting. All captured events were adjudicated as described in Section 2.3.1.

### 2.5 Statistical analysis

All statistical analyses were performed using SPSS version 29.0 and R software (version 4.2.2). Differences in baseline characteristics between groups were analyzed using the chi-square test for categorical variables and the independent samples t-test for continuous variables. A two-sided P-value ≤0.05 was considered statistically significant.

To address potential confounding factors resulting from non-randomized group assignment, we performed propensity score matching (PSM). The propensity score represents the probability of being prescribed low-dose rivaroxaban and was estimated using a logistic regression model. This model included the following covariates: age, sex, history of bleeding, HAS-BLED score, CHA_2_DS_2_-VASc score, estimated glomerular filtration rate (eGFR), and comorbidities (hypertension, diabetes, coronary artery disease, and heart failure). We used a 1:1 nearest-neighbor matching algorithm with a caliper width of 0.2 standard deviations of the logit of the propensity score to create a matched cohort. After matching, balance between the two groups was assessed using standardized mean differences, with all covariates achieving a value of <0.1, indicating good balance.

The incidence of clinical events was compared using the chi-square test or Fisher’s exact test, as appropriate. Time-dependent differences in clinical outcomes between the two groups were evaluated using Kaplan–Meier survival curves and log-rank tests to assess cumulative thromboembolic events, cumulative bleeding events, and composite event rates. Patients who died from non-cardiac causes or completed follow-up without an event were censored at the time of death or last follow-up.

Multivariate logistic regression analysis was conducted to identify independent predictors of rivaroxaban dose selection. The model adjusted for clinically relevant variables and those with P < 0.1 in univariate analysis, including age, sex, body weight, CHA_2_DS_2_-VASc score, HAS-BLED score, estimated glomerular filtration rate (eGFR), history of bleeding, and key comorbidities (hypertension, diabetes mellitus, coronary artery disease) and concomitant medications (use of antiplatelet agents, proton pump inhibitors). For all comparative analyses, effect sizes including Odds Ratios (ORs) for logistic regression and Hazard Ratios (HRs) for time-to-event analyses are reported alongside their 95% Confidence Intervals (95% CIs). Statistical significance was defined as a two-sided P-value <0.05. As a retrospective observational study, a formal prospective sample size calculation was not performed. However, a *post hoc* power analysis was conducted using G*Power software, which indicated that the final sample of 214 patients provided >80% power (α = 0.05) to detect a medium effect size (w = 0.3) in the primary multivariate logistic regression model.

## 3 Results

### 3.1 Baseline characteristics of patients in different rivaroxaban dose groups

A total of 214 elderly patients met the inclusion criteria, comprising 129 males (60.2%), with a mean age of 76.93 ± 8.35 years. Among them, 130 patients received 15–20 mg/day (standard-dose group), and 84 patients received 10 mg/day (low-dose group). The low-dose group had a significantly higher mean age compared to the standard-dose group (79.2 ± 8.2 vs. 75.4 ± 8. years, P = 0.001), and a higher proportion of patients with a history of bleeding (21.42% vs. 7.79%, P = 0.004). No significant differences were observed between the groups regarding sex distribution, CHA_2_DS_2_-VASc score, HAS-BLED score (median = 3, P = 0.586), prevalence of chronic kidney disease, comorbidities, or concomitant medication use (Table 1).

**TABLE 1:** Baseline characteristics of patients by rivaroxaban dose group.

Clinical characteristic	Total n (%)	Low-dose group n (%)	Standard-dose group n (%)	P-value
		Rivaroxaban10 mg/day	Rivaroxaban 15-20 mg/day	
	214	84 (39.2)	130 (60.8)	
Sex				0.298
Man (%)	129(60.2)	47 (55.95)	82 (63.08)	
Female (%)	85(39.72)	37 (44.05%)	48 (36.92%)	
Age (m ± s)	76.93±8.35	79.2±8.2	75.4±8.0	0.001
CHA_2_DS₂-VASc Score	3 (3,4)	3 (3,4.25)	3 (3,4)	0.783
2-3 Score		43 (51.19)	78 (60)	
≥4 Score		40 (47.62)	52 (40)	
HAS-BLED Score	3 (2,3)	3 (2,3)	3 (2,3)	0.586
0-2 Score		36 (42.86)	62 (47.69)	
≥3 Score		48 (57.14)	68 (52.31)	
Comorbidities				
Hypertension (%)	162 (75.7)	62 (73.81)	100 (76.92)	0.604
Diabetes (%)	82 (38.32)	31 (36.9)	51 (39.23)	0.733
Coronary Artery Disease (%)	64 (29.91)	25 (29.76)	39 (30)	0.97
Heart failure (%)	86 (40.19)	33 (39.29)	53 (40.77)	0.829
Ischemic Stroke/TIA	141 (65.89)	52 (61.9)	89 (68.46)	0.323
Chronic Kidney Disease (%)	42 (19.63)	17 (20.24)	25 (19.23)	0.856
Acute Liver Injury (%)	2 (0.93)	2 (2.38)	0 (0)	0.153
Peripheral Artery Disease (%)	33 (15.42)	13 (15.48)	20 (15.38)	0.986
COPD (%)	19 (8.88)	7 (8.33)	12 (9.23)	0.822
Prior Bleeding History (%)	28 (13.08)	18 (21.42)	10 (7.79)	0.004
Concomitant Medications				
Antiplatelet Agents	100 (46.72)	40 (47.61)	60 (46.15)	0.834

### 3.2 Comparison of clinical outcomes between different rivaroxaban dose groups

Composite Endpoint: In the standard-dose group, six patients (4.6%) died, compared to 5 patients (5.9%) in the low-dose group. The difference in all-cause mortality was not statistically significant (P = 0.732).

Primary Efficacy Endpoint: At the 1-year follow-up, 20 patients (15.38%) in the standard-dose group experienced thromboembolic events, compared to 16 patients (19.0%) in the low-dose group. The difference in thromboembolic event rates between the groups was not statistically significant (HR = 1.13; 95% CI: 0.83–1.56; P = 0.532). The incidence density was 16.8 per 100 person-years in the standard-dose group and 21.2 per 100 person-years in the low-dose group.

Primary Safety Endpoint: Two cases of intracranial hemorrhage occurred in the standard-dose group, while no cases were observed in the low-dose group. This difference was not statistically significant (P = 0.498).

Secondary Safety Endpoint: In the standard-dose group, there were 20 bleeding events (15.38%), including 9 gastrointestinal bleeds and 1 case of hematuria. In the low-dose group, 9 bleeding events (10.71%) were reported, including 5 gastrointestinal bleeds. The overall difference in bleeding events between groups was not statistically significant (P = 0.321).

Secondary Endpoint: Unplanned cardiovascular-related readmissions occurred in 26 patients (20%) in the standard-dose group and 23 patients (27.3%) in the low-dose group. The difference in readmission rates was not statistically significant (P = 0. 179) (Table 2).

**TABLE 2:** Comparison of clinical outcomes between rivaroxaban dose groups.

Clinical outcomes	Low-Dose group (n = 84) 10 mg/day	Standard-Dose roup (n = 130) 15-20mg/d	*P*-value
All-cause mortality, n(%)	5 (5.95)	6 (4.61)	0.732^†^
Thromboembolic events n(%)	16 (19.04)	20 (15.38)	
Ischemic stroke/TIA	9	8	0.317*
Angina/Myocardial infarction	7	12	0.536*
Bleeding events,n(%)	13 (15.47)	22 (16.92)	
Intracranial hemorrhage	0	2	0.509^†^
Gastrointestinal bleeding	5	9	0.819*
Hematuria	0	1	1.000^†^
Hemoptysis/Blood-tinged sputum	2	2	0.640^†^
Gum/Nose bleeding	4	5	0.738^†^
Skin/Mucosal ecchymosis	2	3	1.000^†^
Unplanned readmission,n(%)	23 (27.38)	26 (20.0)	0.179*
Laboratory threshold:n(%)	2 (2.38)	8 (6.15)	
Hemoglobin <110 g/L(female), <120 g/L(male)	2	8	0.315^†^

Statistical notation: *, Chi-square test; ^†^, Fisher's exact test.

### 3.3 Comparison of Cumulative Event Rates and survival rates between the two groups

The Kaplan-Meier analysis, which accounts for varying follow-up times and censoring, provided a more nuanced comparison of time-to-event outcomes between the groups.

**FIGURE 2:**
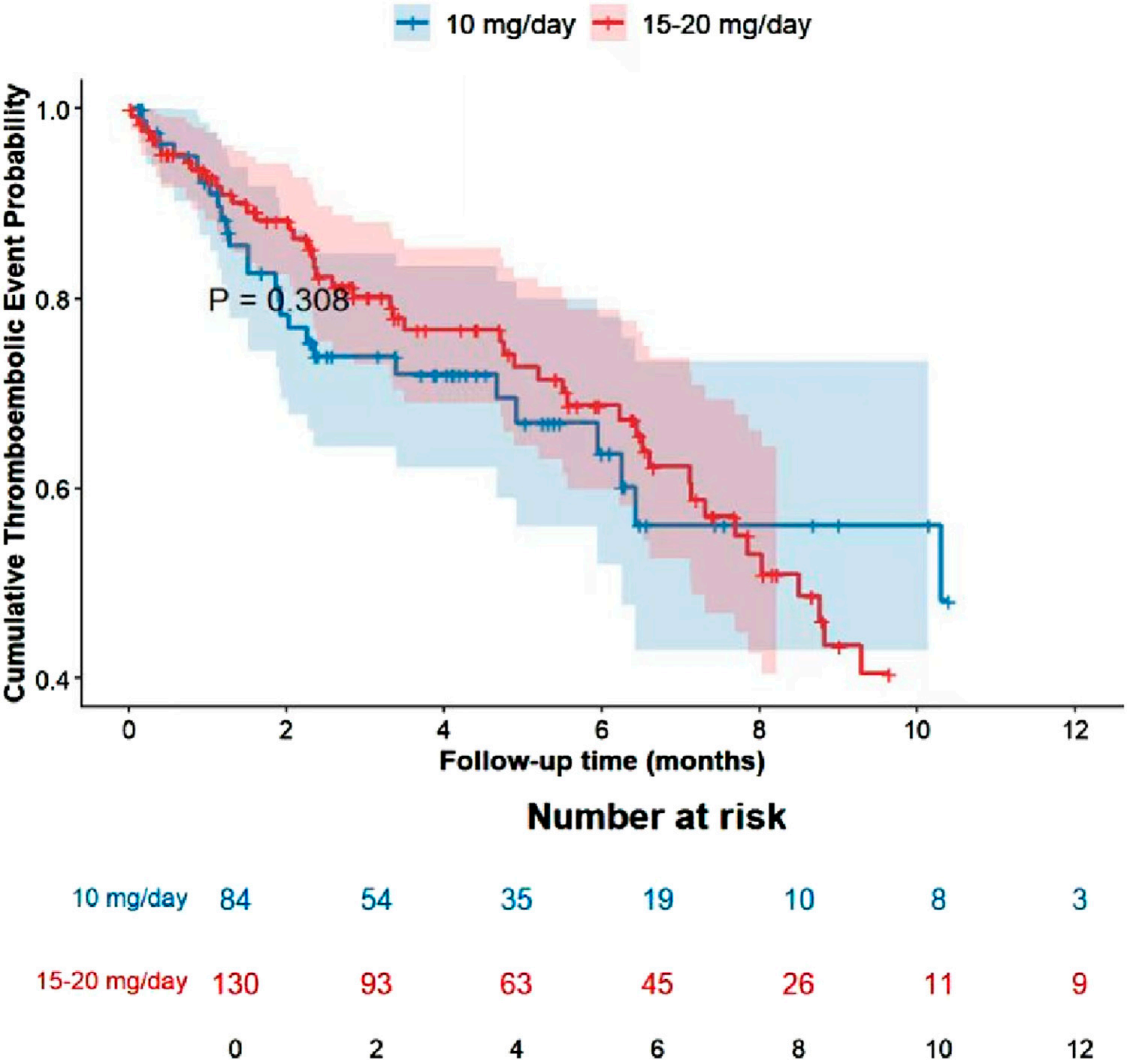
Cumulative incidence of thromboembolic events.

**FIGURE 3:**
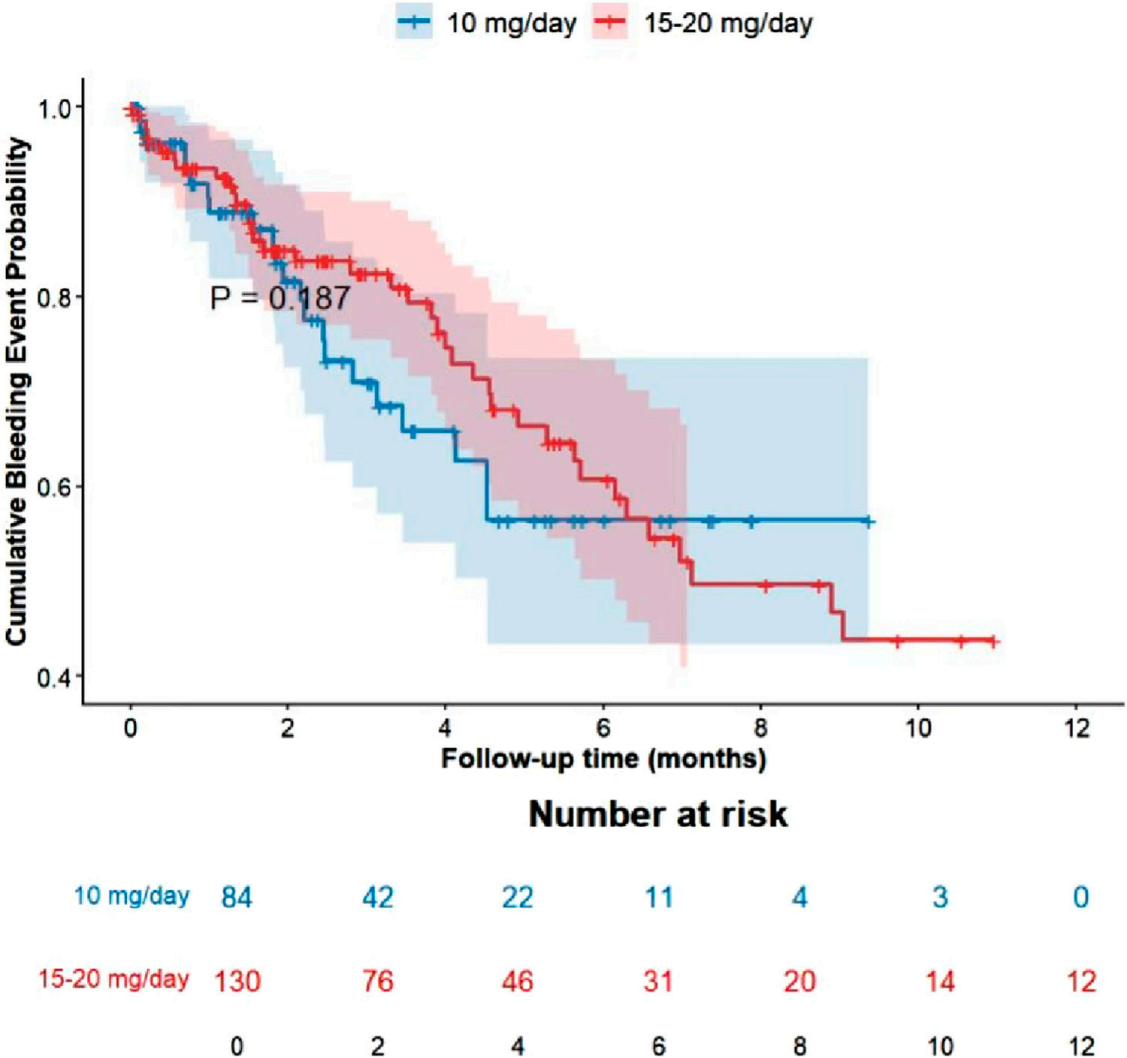
Cumulative incidence of bleeding events.

Cumulative Event Rates: During the 1-year follow-up period, no statistically significant differences were observed in the cumulative incidence of thrombotic events (P = 0.308) or bleeding events (P = 0. 187) between the two groups. The overall trends of the event curves for the standard-dose group (15–20 mg/day) and the low-dose group (10 mg/day) were similar, with no evident dose-dependent differences (Figures 2,3).

**FIGURE 4:**
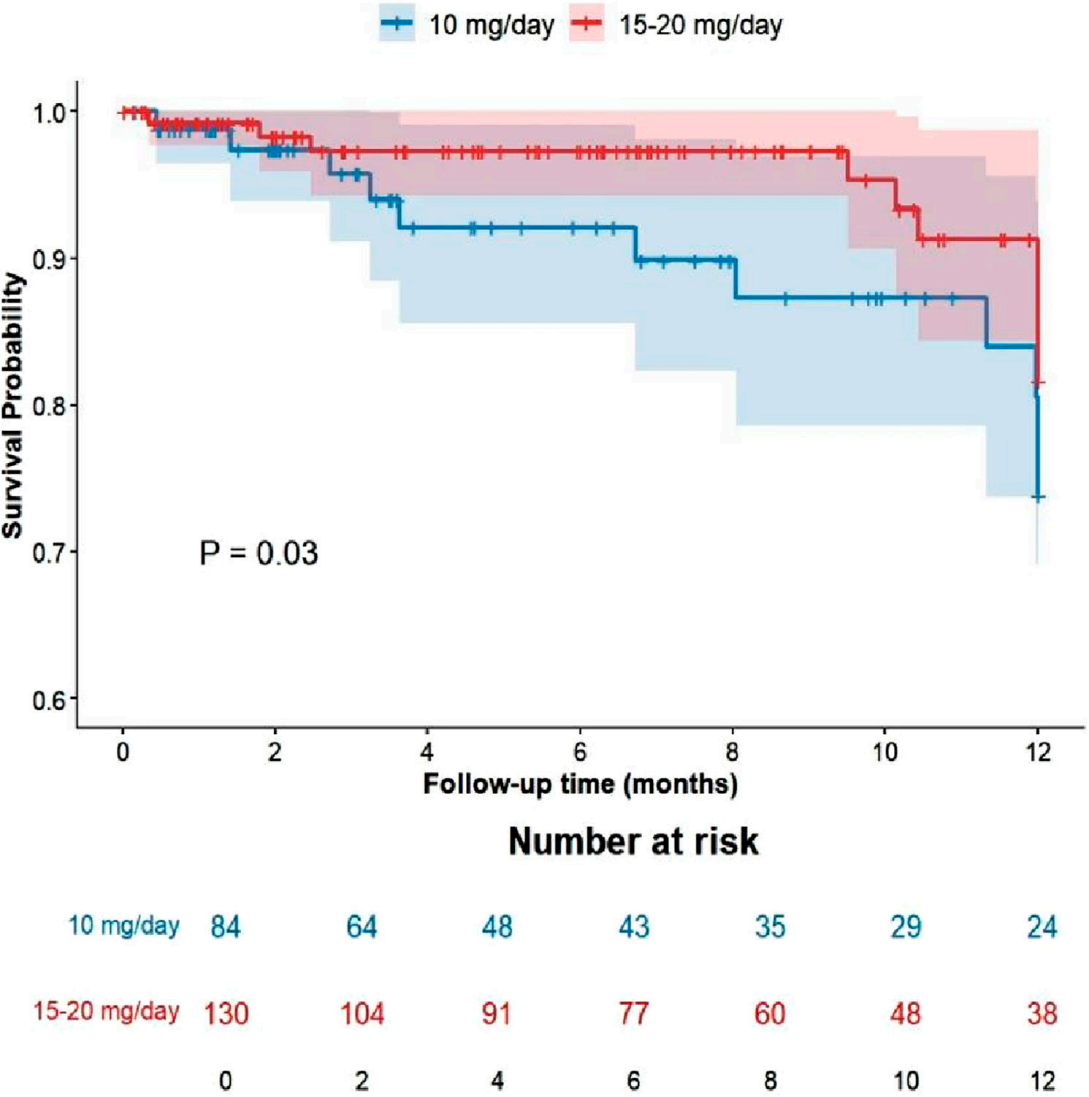
Cumulative survival rates by rivaroxaban dose group.

One-Year Survival Rate: Within the 1-year follow-up, the standard-dose group (15–20 mg/day, n = 130) demonstrated a higher survival rate than the low-dose group (10 mg/day, n = 84), with a statistically significant difference. Although the all-cause mortality rates did not differ significantly between the two groups, the cumulative survival rate at 12 months was significantly higher in the standard-dose group compared to the low-dose group (HR = 0.61; 95% CI: 0.39–0.95; log-rank; P = 0.03) (Figure 4). This Kaplan-Meier estimate accounts for the entire follow-up period and censoring, hence differing from the simple proportion of deceased patients reported in Section 3.2. However, after propensity score matching to balance baseline characteristics, the difference in cumulative survival between the standard-dose and low-dose groups was no longer statistically significant (adjusted HR = 0.75; 95% CI: 0.48–1.18; P = 0.21).

### 3.4 Multivariate analysis of factors affecting rivaroxaban prescription

**FIGURE 5:**
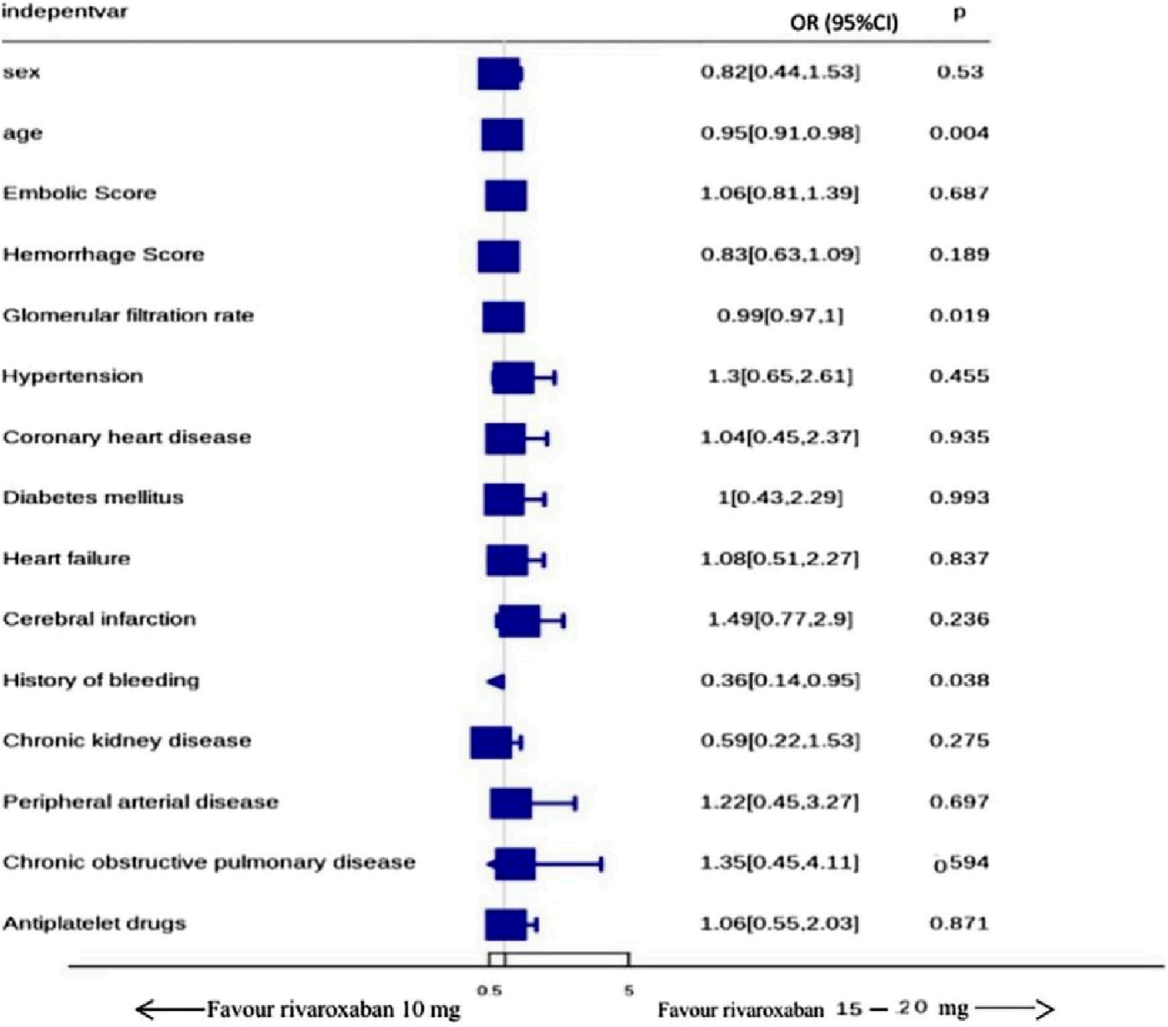
Predictors of low-dose rivaroxaban prescriptio indepentvr.

Binary multivariate logistic regression analysis identified age (OR = 0.95, 95% CI: 0.91–0.98, P = 0.004) and a history of bleeding (OR = 0.36, 95% CI: 0.14–0.95, P = 0.038) as independent predictors for the prescription of low-dose rivaroxaban. For each additional year of age, the likelihood of being prescribed a low dose decreased by 5%. Patients with a history of bleeding were 64% less likely to be prescribed a low dose. Additionally, the eGFR (OR = 0.99, 95% CI: 0.97–1.00, P = 0.019) was also statistically significant, suggesting that patients with impaired renal function were more likely to receive a low-dose regimen. Other variables, including gender, embolic score, bleeding score, hypertension, coronary artery disease, diabetes, and heart failure, were not significantly associated with rivaroxaban dosage selection (all P > 0.05) (Figure 5). Thus, the decision to prescribe the low-dose regimen was primarily driven by older age, a history of bleeding, and impaired renal function.

## 4 Discussion

This single-center, retrospective study of 214 elderly Chinese patients with NVAF yielded two principal findings. First, the low-dose regimen of rivaroxaban (10 mg/day) was not associated with significantly different rates of thromboembolic (15.38% vs. 19.0%, P = 0.308) or bleeding (15.38% vs. 10.71%, P = 0.187) events compared to the standard dose (15–20 mg/day). Notably, this comparable efficacy and safety profile was observed in the low-dose group, even though they had a significantly less favorable baseline risk profile characterized by older age and a higher prevalence of prior bleeding, both of which are established predictors of adverse outcomes. These results suggest that the empirical use of a low-dose regimen in select frail elderly patients may be a reasonable clinical strategy within this real-world clinical setting. This approach may effectively balance thrombotic and bleeding risks, potentially achieving a comparable net clinical benefits in a patient subgroup predisposed to worse outcomes.

Second, the standard-dose group exhibited a higher 12-month cumulative survival rate in the unadjusted analysis (HR = 0.61; 95% CI: 0.39–0.95; log-rank; P = 0.03). However, it is important to note that this apparent difference was substantially reduced and lost statistical significance after adjusting for baseline confounders using propensity score matching and multivariable analysis. This pattern strongly suggests that the initial finding was confounded by the higher-risk profile of the low-dose group. The non-randomized design precludes causal inference; the observed survival difference is most plausibly explained by pre-existing disparities in baseline risk profiles between the groups, i.e., confounding by indication, rather than a direct therapeutic benefit of the standard rivaroxaban dose. Clinicians preferentially prescribed the lower dose to patients perceived as frailer and at higher mortality risk, thereby systematically creating a “negatively selected” low-dose cohort with a higher underlying risk of mortality.

Our study must be interpreted in the context of its observational design and the inherent limitation of confounding by indication. Our multivariate analysis confirmed that clinical factors, such as advanced age, history of bleeding, and impaired renal function, were key determinants in dose selection. Consequently, the two dose groups were not directly comparable at baseline. Thus, the observed differences in outcomes, including the initial disparity in cumulative survival, are more likely to reflect underlying differences in clinical risk profiles than a direct causal effect of the rivaroxaban dose itself.

In addition to clinical factors, inter-individual genetic variability is another important source of differences in rivaroxaban response. Specifically, polymorphisms in genes that encode drug transporters and metabolizing enzymes, such as ABCG2 (BCRP) and CYP3A4/5, can significantly alter rivaroxaban’s pharmacokinetics. The allele frequencies of these functional variants, particularly the ABCG2 c.421C>A polymorphism, are higher in Asian populations. This may contribute to the real-world prescribing patterns observed and partly explain why lower doses were equally effective and safe in our cohort. The lack of pharmacogenomic data is a recognized limitation of our study and of many current retrospective studies. However, this limitation highlights a critical avenue for future research in personalized anticoagulation therapy.

Nevertheless, our central finding of comparable event rates in a higher-risk population offers a compelling perspective on the ongoing debate regarding low-dose rivaroxaban. Our results align with those from the J-ROCKET AF trial and others, which reported similar safety and efficacy between doses. They are also consistent with real-world studies by Briasoulis et al. (2020). More importantly, our findings may reconcile conflicting results in the literature. For example, the Taiwanese study by Cheng WH et al. (2019) observed an elevated non-ST-segment elevation myocardial infarction risk in low dose, which may have been due to the inclusion of a particularly high-risk subgroup. This underscores the fact that the critical question is not merely “low dose versus standard dose,” but rather, “For which patient profile does a low-dose strategy offer the optimal risk-benefit balance?” Overall, our findings contribute to shifting the discussion from a simple dose contrast paradigm to a more refined, risk-stratified anticoagulation framework.

The prevalent use of the 10 mg dose in our cohort (39.3%) and across Asia is further supported by emerging data (Chan YH et al., 2023; Yan Q, et al., 2025). Pharmacokinetic studies demonstrate that Chinese patients achieve drug levels comparable to those of Western populations on 15 mg with 20 mg (Ng et al., 2018) Coupled with the high frequency of ABCG2 loss-of-function polymorphisms in Asians (Xiang Q et al., 2023), which may increase drug exposure and bleeding risk, this provides a pharmacological rationale for this practice (Guo LF et al., 2025; Wu T et al., 2023; Wang Y et al., 2021). However, the creatinine clearance–based dose adjustment strategy currently in use has recognized limitations, including its static nature and inability to fully accommodate nonrenal metabolism and pharmacogenomic variability.

There are several important limitations of our study that must be acknowledged. This study has several limitations. First, the single-center, retrospective design and exclusive inclusion of hospitalized patients may have introduced selection bias, limiting the generalizability of the findings. As demonstrated by Lee PT et al. (2025) hospitalized AF patients tend to be older, exhibit greater disease severity, and have a higher burden of clinical comorbidities compared to out patients (Lee PT et al., 2025) and community-dwelling patients. Our study population is characterized by this context, implying that our findings are primarily applicable to this high-baseline-risk subgroup. Conclusions should be extrapolated with caution to the broader population of stable, community-based elderly AF patients. These factors may affect the generalizability of our findings to healthier, community-dwelling elderly populations. Second, the follow-up period was limited. Third, renal assessment relied solely on eGFR. Additionally, the absence of pharmacogenomic data precludes analysis of how genetic variations may influence outcomes. Finally, the absence of body weight data is a limitation; however, population pharmacokinetic studies have demonstrated that body weight has only a limited, clinically insignificant impact on rivaroxaban exposure (Mausteller KG et al., 2022; Ashton V et al., 2021).

In conclusion, this exploratory analysis suggests that low-dose rivaroxaban is a viable, risk-adapted alternative for vulnerable elderly patients in real-world clinical practice, such as those who are very elderly or have a significant bleeding history. Based on our observational data, standard-dose rivaroxaban can be considered for elderly patients with NVAF and stable renal function and low bleeding risk. The low-dose regimen is a reasonable option for patients aged ≥80 years, with high bleeding risk, or with declining renal function. By elucidating the clinical rationale behind empirical dose adjustments, our work establishes a foundation for implementing individualized anticoagulation strategies. Ideally, future prospective studies would integrate ambulatory monitoring, pharmacogenomics, and standardized frailty assessments to validate these findings and further refine personalized dosing regimens.

## Data Availability

The original contributions presented in the study are included in the article/Supplementary Material, further inquiries can be directed to the corresponding author.
